# Recruitment of Older Adults During the COVID-19 Pandemic: Utilizing Two Recruitment Techniques

**DOI:** 10.1093/geroni/igab046.810

**Published:** 2021-12-17

**Authors:** Martha Coates, Justine Sefcik, Zachary Hathaway, Rose Ann DiMaria-Ghalili

**Affiliations:** 1 Drexel University, Bryn Mawr, Pennsylvania, United States; 2 Drexel University, College of Nursing and Health Professions, Philadelphia, Pennsylvania, United States; 3 Drexel University, Philadelphia, Pennsylvania, United States

## Abstract

The COVID-19 pandemic has limited in-person interactions and reduced access to research participants. To recruit older adults for a study on the impact of COVID-19 on physical, mental, and social wellbeing we utilized two recruitment techniques: 1) ResearchMatch, a free recruitment database, and 2) a convenience sample of residents in a retirement community. Messages were sent via ResearchMatch to 1,491 adults age 65 and over. In total, 228 individuals responded over 2 weeks; 194 responded in the first 24 hours. Eighty-four completed the online survey. For the retirement community, recruitment information was shared during a Zoom townhall meeting; 44 expressed interest and 30 completed the study (half over the phone with a research assistant). We will discuss differences between the older adults recruited by each strategy (e.g., the ResearchMatch group was highly educated; more staff needed to interview retirement community participants). Overall, these were effective recruitment techniques during challenging times.

